# Clinical Management of Obsessive-Compulsive Disorder in Children and Young People: Evaluating Current Practices in Al Ain, United Arab Emirates

**DOI:** 10.1192/bjo.2025.10490

**Published:** 2025-06-20

**Authors:** Amani Alkharoossi, Ayesha Afzal, Syed Fahad Javaid

**Affiliations:** 1 Behavioral Sciences Institute, Al Ain Hospital, Al Ain, UAE; 2 Department of Psychiatry, College of Medicine and Health Sciences, United Arab Emirates University, Al Ain, UAE

## Abstract

**Aims:** Obsessive-compulsive disorder (OCD) is a condition characterised by remitting and relapsing symptoms that can be debilitating, significantly impacting a young person’s daily life. Individuals with this condition experience distressing symptoms that include obsessions in the form of repetitive, intrusive thoughts and compulsions manifested as persistent rituals. All children and young people with OCD should be offered guided self-help, psychological support, and pharmacological treatment options tailored to the patient’s developmental age. This audit aimed to evaluate the clinical management of OCD in children and adolescents at the Behavioural Science Institute, Al Ain Hospital, United Arab Emirates. We analysed compliance with the standards set forth in The National Institute for Health and Clinical Excellence (NICE) Guideline 31 regarding the diagnosis and management of OCD in children and young people.

**Methods:** This hospital-wide audit involved a retrospective review of electronic case notes. A questionnaire was developed to anonymously capture the necessary information. The audit sample consisted of 39 service users diagnosed with OCD who were treated in the child psychiatry clinic between January 2019 and December 2023. Data collection occurred between April and June 2024.

**Results:** Of the 39 patients, 20 (51%) were male, with 18 (46%) being Emirati citizens. The age range of the sample was between 7 and 15 years, with a mean age of 9.4 years. Among the 39 patients, only one received clear, guided self-help materials in the form of access to an interactive app for breathing exercises and relaxation strategies. Thirty-two (82%) of the patients received psychotherapy, with the number of sessions ranging from 1 to 28. Psychotropic medications were administered to 28 (72%) of the patients, all treated with a selective serotonin reuptake inhibitor (SSRI). Sertraline was the most commonly prescribed medication, followed by fluoxetine and fluvoxamine.

**Conclusion:** This audit has identified areas for improvement in the current practice of treating OCD among children and adolescents, including the need to develop local guidelines, increase access to self-help materials for patients, and enhance the services provided for psychotherapeutic interventions. We recommend improved staff training to enhance the quality of discussions with the young person and their family, which may help increase compliance with psychotherapy. A re-audit of the practice will be conducted one year after the implementation of the aforementioned action plan.

No financial sponsorship has been received for this evaluative exercise.

